# From single-cell atlases to predictive intercellular response networks in liver cancer

**DOI:** 10.1016/j.mmr.2026.100076

**Published:** 2026-10-27

**Authors:** Ralf Weiskirchen

**Affiliations:** Institute of Molecular Pathobiochemistry, Experimental Gene Therapy and Clinical Chemistry, RWTH University Hospital Aachen, D-52074 Aachen, Germany

**Keywords:** Liver cancer, Hepatocellular carcinoma, Single-cell RNA sequencing, Spatial transcriptomics, Tumor microenvironment, Intercellular communication, Precision oncology

Cancer research has long been driven by the search for altered genes, deregulated pathways, and molecular subclasses. In liver cancer, this perspective has been highly productive, but it is no longer sufficient. Liver cancer usually develops in chronically diseased tissues shaped by inflammation, fibrosis, cirrhosis, metabolic stress, vascular remodeling, and immune adaptation. Therefore, the decisive biological question is increasingly not only which pathway is altered, but where, in which cell type, through which intercellular communication mechanisms, and under which therapeutic pressure it becomes clinically relevant. The bibliometric analysis by Chen *et al.* [1] provides a useful overview of this transition, in which single-cell RNA sequencing (scRNA-seq) has rapidly become a central technology in liver cancer research, with an emerging emphasis on the tumor microenvironment, immunotherapy, prognosis, spatial transcriptomics, angiogenesis, machine learning, and multi-omics integration.

scRNA-seq has changed liver cancer biology by assigning transcriptional programs to malignant, immune, endothelial, fibroblast, and progenitor-like compartments rather than averaging them across mixed bulk tissue [2], [3]. Yet the field should resist the temptation to equate higher resolution with deeper understanding. A catalog of cell types, however refined, does not automatically explain tumor behavior. The next step must be to determine how these cell populations communicate, adapt, compete, and respond to treatment.

A clinically meaningful single-cell finding should be interpreted as part of a response network. Such a network includes the cell population in which a pathway is active, the upstream ligand-receptor signals that sustain it, the downstream transcriptional programs that execute it, the tissue niche in which it occurs, and the clinical context in which it matters. This concept is consistent with broader tumor-atlas initiatives that aim to chart cancer transitions across space and time at single-cell resolution [4]. For liver cancer, it provides a practical framework to connect cellular heterogeneity with prognosis, immune escape, angiogenesis, invasion, and therapy resistance.

This shift has immediate relevance for well-known pathways. WNT/β-catenin signaling, for example, should not be interpreted solely as a tumor-intrinsic alteration. Its therapeutic meaning may differ depending on whether it marks immune exclusion, progenitor-like malignant plasticity, or crosstalk with stromal and endothelial compartments. Similarly, vascular endothelial growth factor (VEGF)-related signaling should not be reduced to angiogenesis alone. It may form part of a vascular-immune circuit that regulates myeloid recruitment, T-cell exclusion, and sensitivity to combined anti-angiogenic and immune-checkpoint therapy. The clinical success of atezolizumab plus bevacizumab illustrates how vascular and immune biology can converge therapeutically in hepatocellular carcinoma (HCC) [5].

Spatial context is indispensable for this interpretation. Liver tumors are not homogeneous cell suspensions, but rather structured ecosystems containing invasive margins, fibrotic septa, hypoxic areas, perivascular zones, immune-excluded tumor nests, and lymphoid-like aggregates. A spatial study in primary liver cancer has already shown that architecture matters for understanding tumor biology [6]. Therefore, spatial transcriptomics and spatial proteomics should not merely describe where cells are located. They should test which niches drive immune escape, which cellular interactions are therapeutically reversible, and which spatial biomarkers can be translated into clinically robust assays.

Immune checkpoint inhibitors and perioperative immunotherapy strategies are reshaping HCC treatment, but durable responses remain limited to subsets of patients [7]. A response-network approach could help distinguish tumors with productive antitumor immunity from tumors with exhausted T-cell accumulation, suppressive myeloid dominance, vascular exclusion, or stromal barriers. These distinctions are not academic. They may guide rational combinations, such as checkpoint blockade with anti-angiogenic therapy, macrophage reprogramming, transforming growth factor-β (TGF-β) inhibition, or pathway-directed targeted therapy. A single-cell study showing endothelial reprogramming and immunosuppressive macrophage induction illustrates how specific cellular circuits can suggest therapeutic vulnerabilities [8].

However, establishing these networks as mechanistic targets for intervention requires causal validation. Computational inference of ligand-receptor interactions and pathway activity is valuable, but it remains hypothesis-generating unless functionally tested. Methods for inferring cell-cell communication from gene expression have matured substantially, but their outputs must be interpreted with biological caution [9]. The field now needs more perturbation experiments using organoids, tumor explants, co-culture systems, clustered regularly interspaced short palindromic repeats (CRISPR)-based screens, lineage tracing, proteomics, and humanized models. The key question is whether an inferred intercellular circuit actually drives tumor growth, immune escape, angiogenesis, invasion, or drug resistance, and whether it can be therapeutically reversed.

Machine learning will be important, but only if it remains interpretable. Predictive models should not become black boxes that classify patients without explaining the underlying biology. Their outputs should be traceable to defined cell types, spatial niches, signaling pathways, and treatment-relevant mechanisms. This is especially important because single-cell data science still faces major challenges, including batch effects, dropout events, tissue dissociation bias, computational complexity, and cross-study comparability [10**]**. Without careful validation and transparent reporting, response-network signatures may remain dataset-specific rather than clinically transferable.

A practical roadmap is therefore needed. First, discovery studies should identify reproducible pathway-defined cell states and communication-defined response states across etiologies, disease stages, and treatment contexts. Second, spatial and multi-omics validation should determine whether these states form biologically meaningful niches and whether transcriptomic activity corresponds to protein expression, chromatin accessibility, ligand-receptor signaling, or functional pathway activation. Third, perturbation studies should test whether the inferred networks are causal. Fourth, simplified clinical assays should be developed from the most robust signatures, including multiplex immunohistochemistry, spatial protein panels, circulating biomarkers, or targeted RNA panels.

Before clinical implementation, the resulting biomarkers should undergo independent, prospective validation in the intended patient population and treatment setting. In conclusion, liver cancer research is entering a phase in which single-cell and spatial technologies can move beyond atlas generation toward predictive precision oncology (Fig. 1). The real value of this transition is not merely higher resolution, but better biological accountability. Pathways can now be interpreted in their cellular, spatial, immune, communicative, and therapeutic context. The challenge ahead is to transform these insights into validated response-network biomarkers, rational combination therapies, and clinically actionable strategies for patients with liver cancer.

**Fig. 1:**
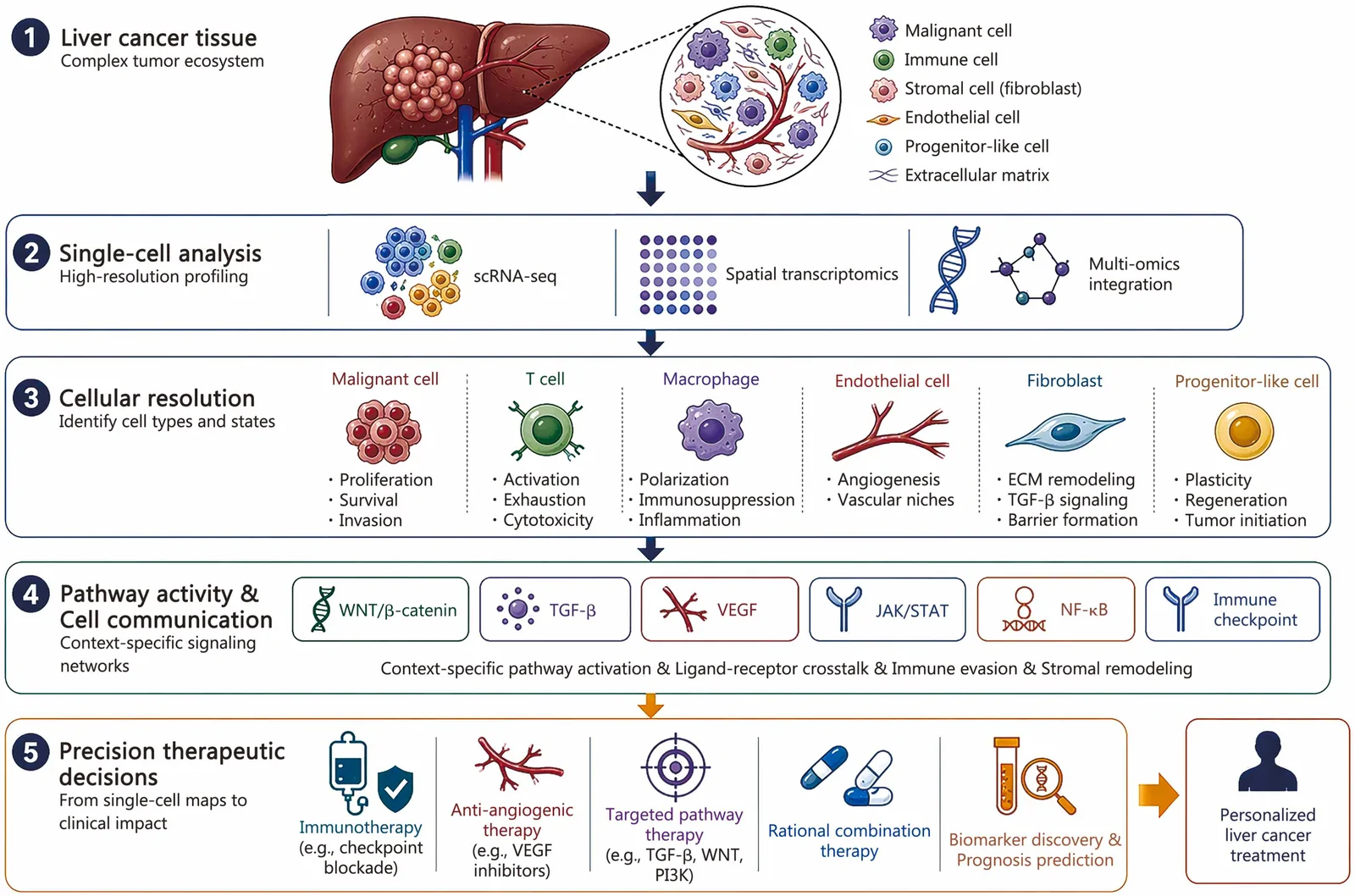
From single-cell profiling to predictive intercellular response networks in liver cancer. A conceptual framework illustrating how single-cell technologies can translate biological insights into clinically actionable strategies for liver cancer. Starting from the complex tumor ecosystem, including malignant, immune, stromal, endothelial, and progenitor-like cell populations, scRNA-seq, spatial transcriptomics, and multi-omics integration provide high-resolution characterization of cellular heterogeneity. These approaches identify cell type-specific signaling programs and support the interference of candidate intercellular response networks involving pathways such as WNT/β-catenin, TGF-β, VEGF, JAK/STAT, NF-κB, and immune-checkpoint signaling. Integration and validation of these molecular insights enables biomarker discovery, patient stratification, rational combination therapies, and personalized treatment approaches based on predicted tumor-immune, vascular-immune, and stromal-tumor response states in hepatocellular carcinoma. ECM. Extracellular matrix; JAK/STAT. Janus kinase/signal transducer and activator of transcription; NF-κB. Nuclear factor κB; PI3K. Phosphoinositide 3-kinases; scRNA-seq. Single-cell RNA sequencing; TGF-β. Transforming growth factor-β; VEGF. Vascular endothelial growth factor; WNT/β-catenin. Wingless-related integration site/β-catenin signaling pathway.

## Abbreviations

HCC: Hepatocellular carcinoma

scRNA-seq: Single-cell RNA sequencing

TGF-β: Transforming growth factor-β

VEGF: Vascular endothelial growth factor

## Ethics approval and consent to participate

Not applicable.

## Author’s contribution

RW conceived the editorial, wrote and revised the manuscript, approved the final version, and takes full responsibility for its content.

## Funding

This work was supported by the German Cancer Aid (70115581). The funder had no role in the conceptualization, writing, or decision to submit this article for publication.

## Competing interests

The author declares that there are no competing interests.

## Data Availability

Not applicable.
